# Pennsylvania

**Published:** 1899-07

**Authors:** 


### SOCIETY PROCEEDINGS.

### PENNSYLVANIA STATE VETERINARY MEDICAL ASSOCIATION.

*Report of W. S. Kooker, V. S., Philadelphia.*

THERE has not been very much accomplished in the way of improving our profession, intellectually or financially, during the year just passed. We worked hard to improve our pay and standing in the army, with very little success. The bill passed the House with recognition, pay, and allowances of second lieutenant, but got lost in the Senate, as were all other proposed additions to the present staff of officers. We should not be discouraged by this failure, but pull and push together until we accomplish that which is our just rights. There were sent out by our hard-working and always-to-be-depended-upon friend (Dr. Hoskins), with the help of two extra stenographers, 7000 letters, of which I suppose that each of you received one, and thought that it came to you from Washington.

Some novel methods of advertising to obtain business during the past years of depression, among certain members of the profession, are to be noted. This "ad" was in the Philadelphia *Record* only a few days ago: "What have you to exchange for veterinary services? Address *Record* office, etc." It affords me great pleasure to announce to you that I succeeded in learning who he was, and that he is no member of any veterinary association. Another method of enlarging a practice, not destined to be a credit to our profession, is the taking of contracts at figures so cheap that it is disgraceful to the profession, and should be discouraged.

It is a suggestion of mine that the county secretaries of the Pennsylvania Veterinary Association should do missionary work in the way of soliciting good, effective workers for members in our Association. They are appointed by our presidents on account of their knowledge of the profession and its practitioners. By activity in this line they will greatly assist in increasing our membership as well as our usefulness.

We have heard some criticisms upon political veterinarians. We think it is the duty of veterinarians, as well as members of other professions, to do their part in the ruling and shaping of our politics. We have had a great honor bestowed upon one of our fellow-members. I refer to our esteemed friend, Dr. W. Horace Hoskins, late candidate for mayor of this city. Our worthy President of this Association has had the honor of the mayoralty of his city. For all of these honors we should feel very proud.

A number of new works have been issued from American veterinary writers, and their general worth is much in advance of those published on similar subjects in the past, and show the tendency to and result of our higher veterinary education in this country.

We are particularly proud of the honors bestowed upon our fellow-members in their appointment to official positions in different States of the Union. We refer to our friends Repp, Shaw, Mohler, Louis A. Klein, Lushington, and H. A. Christman.

*Report of Otto G. Noack, V.S., of the Committee on Sanitary Science and Police.*

In compliance with your request, I will state that during the last year contagious diseases were not quite so prevalent in this section as before. A year ago there were many cases of cholera and diphtheria in fowls, but this year none were brought to my notice.

Hog-cholera with its destructive consequences did not reach this county, and only from one farmer I heard two pigs had died from this disease.

Rabies has only known to be in existence in some cases in dogs which have been killed, but not before they had bitten some people. I did not hear with what after-effect, as the wounds had been cauterized immediately, and the definite result did not come to my knowledge. I think that if there could be some compulsory law to chain the dogs during the months of August to November, or the owners be compelled to muzzle their dogs, the fear for rabid dogs would disappear and not every dog that bites would be called rabid.

Tuberculosis is still in existence, but the farmer, becoming more educated, is not, as he used to be, hostile toward the execution of the law; on the contrary, he is seeking the aid of the State and looking at the laws in this direction as a good thing.

Croupous pneumonia in cattle in the last six months came quite often under my observation. Iufluenza and strangles among horses were in existence in the same degree as in the past. One case of pyæmia in a mule, with deadly result, came under my care. One case of diabetes insipidus was treated with a better effect.

Azoturia followed quite frequently after the heavy snow-storms of February, the horses being confined, with good food, to the stables during this period, and did not come in contact with the bacilli of this disease, which the French authors claim to be the cause.

Mange and distemper in dogs were raging just the same as in the past. Some cases of acarus were brought to me for treatment.

At last I will call your attention—overlooking the bad meat and milk supply in this municipality without any regulations for the better—to the rules in Switzerland for the suppression of the sale of patent medicines, where same can only be sold with the permission of the Cantonal Sanitary Boards—a good move, in which our government should join.

*Report of Dr. W. L. Rhoads, Corresponding Secretary.*

My annual report as Corresponding Secretary of your Association for the year ending March 7, 1899, is as follows:

| | |
|---|---|
| Balance from report of March 8, 1898 . . | $ 71.05 |
| Moneys received as semi-annual dues . . | 64.00 |
| Moneys received from new members . . . | 50.00 |
| Total received, March 7, 1899 . . . | $185.05 |

EXPENDITURES.

| | |
|---|---|
| Paid Avil & Co. for printing, bill of March 1, 1898 | $ 15.00 |
| Paid Charles A. Dixon Printing House for printing to date, as per bill of February 27, 1899 (attached) . . . . . . . | 99.35 |
| Engrossing twenty-one certificates at 20 cts. each, | 4.20 |
| Postage, etc., up to and including semi-annual meeting . . . . . . . . . . | 24.00 |
| Postage, etc., for semi-annual meeting up to and including notices for annual meeting . . | 20.50 |
| Paid Dr. Bridge (Treasurer) check for balance in my hands March 6, 1899 . . . . . | 22.00 |
| Amount paid out to March 7, 1899 . . | $185.05 |

There were 300 programmes sent out for our semi-annual meeting in Pittsburg to the veterinarians of Pennsylvania and adjoining States, in response to which we had probably the best semi-annual meeting ever held by the Association.

There is, I trust, a copy of our new by-laws, etc., in the hands of every member of our Association, also in the hands of every veterinarian throughout the State who is interested in veterinary progression. There were 1000 programmes sent to the veterinarians of this and adjoining States in the interest of our present meeting, with what results you can judge for yourselves.

As Corresponding Secretary, I take this opportunity of urging our members to assist the officers in the increase of membership, and, by each doing missionary work in his own district, endeavor to bring in at least one desirable member at each meeting; by so doing we will soon have an organization that will be asked what legislation we would like to have, instead of begging for that which we should have had years ago.

Now, brother members, those of you who have not done so, comply with the by-laws and pay your dues in advance, as though it was a pleasure for you to part with the filthy lucre in such a good cause. You will thus gain pleasure, and the Association will profit thereby.

I now wish to thank those who have so willingly assisted me in my work during the past year.

